# Protein phosphatase 4 maintains the survival of primordial follicles by regulating autophagy in oocytes

**DOI:** 10.1038/s41419-024-07051-4

**Published:** 2024-09-08

**Authors:** Ming-Zhe Dong, Ying-Chun Ouyang, Shi-Cai Gao, Lin-Jian Gu, Jia-Ni Guo, Si-Min Sun, Zhen-Bo Wang, Qing-Yuan Sun

**Affiliations:** 1 State Key Laboratory of Stem Cell and Reproductive Biology, Institute of Zoology, Chinese Academy of Sciences, Beijing, China; 2 University of Chinese Academy of Sciences, Beijing, China; 3 Fertility Preservation Lab, Guangzhou Key Laboratory of Metabolic Diseases and Reproductive Health, Guangdong-Hong Kong Metabolism & Reproduction Joint Laboratory, Reproductive Medicine Center, Guangdong Second Provincial General Hospital, Guangzhou, China

**Keywords:** Infertility, Oogenesis

## Abstract

In mammalian ovary, the primordial follicle pool serves as the source of developing follicles and fertilizable ova. To maintain the normal length of female reproductive life, the primordial follicles must have adequate number and be kept in a quiescent state before menopause. However, the molecular mechanisms underlying primordial follicle survival are poorly understood. Here, we provide genetic evidence showing that lacking protein phosphatase 4 (PPP4) in oocytes, a member of PP2A-like subfamily, results in infertility in female mice. A large quantity of primordial follicles has been depleted around the primordial follicle pool formation phase and the ovarian reserve is exhausted at about 7 months old. Further investigation demonstrates that depletion of PPP4 causes the abnormal activation of mTOR, which suppresses autophagy in primordial follicle oocytes. The abnormal primordial follicle oocytes are eventually erased by pregranulosa cells in the manner of lysosome invading. These results show that autophagy prevents primordial follicles over loss and PPP4-mTOR pathway governs autophagy during the primordial follicle formation and dormant period.

## Introduction

In mammals, the duration of fertility of a female is determined by the initial size of primordial follicle pool and by the rate of its activation and depletion [1]. Primordial follicles are formed from embryonic day 17.5 (E17.5) to postnatal day 5 (PD5) in mice [2, 3], during which oocyte cyst breaks down and individual oocytes are surrounded by flat granulosa cells (pre-granulosa cells). At PD5, a fixed stockpile of primordial follicles, about 2500–6000, are typically observed in the ovary and about two-thirds of oocytes are lost during the cyst breakdown process [3]. These survival primordial follicles can have three possible fates: A, they remain quiescent (not growing but surviving); B, limited numbers of them are continuously activated, mature, and undergo ovulation; C, they die out progressively and are cleared directly from quiescence or after activation [4]. The three courses maintain a balance under indefinite mechanisms and disorders of any course could causes primary ovarian insufficiency (POI) or premature ovarian failure (POF). When the available pool of primordial follicles has been exhausted, ovulation ceases and women enter menopause [5, 6].

Several possible mechanisms, including apoptosis [7, 8], autophagy [9, 10], and the direct extrusion from the ovarian surface [3, 11] have been proposed for explaining oocyte loss during primordial follicle formation. However, the reason and regulators behind them remain unknown. Several studies hold different perspectives which suggested that prepubertal primordial follicle loss in mice is not due to classical apoptotic pathways, because the ensuring death of the cell did not exhibit the morphological signs of apoptosis [12, 13]. Recent investigations uncover that autophagy plays an important role in the maintenance and regulation of ovarian primordial follicle reserve [14], anti-ovarian aging [15, 16], and even differentiation of ovarian granulosa cells [17]. Activation of autophagy in early neonatal mice could increase primordial follicle number and improve lifelong fertility [10]. It is well known that mTOR (mechanistic target of rapamycin kinase) and autophagy have an inverse relationship [18]. Inhibition the mTOR pathway before PD5 could induce autophagy, which further encouraged the gathering of primordial follicles and the existence of oocytes [19]. However, complete deletion of mTOR in mouse oocytes caused progressive degeneration of primordial follicle oocytes and loss of granulosa cells with age [20]. anti-Mullerian hormone produced by pregranulosa cells also takes part in protecting primordial follicle pool via inducing autophagy in ovaries by inhibiting FOXO3/FOXO3A phosphorylation in the case of follicle depletion [21]. Germ cell-specific knockout of autophagy-related genes, including *Atg7* [22], *Atg9a* [23], and *Epg5* [24], lead to POI with the decrease of follicle as well as oocyte number in the ovary, which point out the crucial role of autophagy during folliculogenesis.

Numerous studies using genetically modified mice reveal that protein kinases play important roles during folliculogenesis. It has been reported that lacking PDK1 in oocytes results in loss of primordial follicles during early adulthood due to suppression of PDK1-AKT-S6K1-rpS6 signaling pathway [25]. We also found that ablation of LKB1 [26] or CK2 [27] caused primordial follicle loss followed by POF. It is generally believed that protein kinases and phosphatases are equally important to balance the phosphorylation status during cellular process. The phosphatase PTEN was reported to govern follicular activation via PI3K, PIP3, AKT, and FOXO3A [1]. Among the serine/threonine phosphoprotein phosphatases (PPPs), PPP2A, PPP4, and PPP6 form a subfamily called PP2A-like protein phosphatases. Our knockout mouse model revealed that oocyte PPP2A is dispensable for folliculogenesis [28], while PPP6 plays an important role for maintenance of primordial follicle pool by safeguarding genomic integrity [29]. PPP4, a member of the subfamily, at least has three distinct complexes containing a catalytic subunit (PPP4C) with different regulatory subunits (PPP4R1, PPP4R2, and PPP4R3α or PPP4R2 and PPP4R3β) [30]. In cell lines, PPP4 has been shown to contribute to DNA damage repair by dephosphorylating γ-H2AX, RPA2, 53BP1, and KAP-1, which regulate the essential steps in DNA damage repair [30–33]. To clarify this gene’s function in vivo, we recently reported that specifical deletion *Ppp4c* from growing follicle oocytes (by crossing *Ppp4c*^*fl/fl*^ mice with *Zp3-Cre* mice) causes defective early embryonic development without causing female POI [34]. However, in the present study, we found that deletion *Ppp4c* in mouse oocytes during the primordial follicle stage (by crossing *Ppp4c*^*fl/fl*^ mice with *Gdf9-Cre* mice) results in accelerated ovarian aging due to the severe loss of primordial follicles, which is caused by suppressed autophagy in oocytes. We further corroborated that depletion of PPP4 causes the abnormal activation of mTOR, which suppresses autophagy in primordial follicle oocytes. Thus, PPP4C-mTOR signaling in oocytes appears to be involved in the maintenance of primordial follicles.

## Results

### *Ppp4c* specific knockout in primordial follicle oocytes results in female infertility

**Fig. 1:**
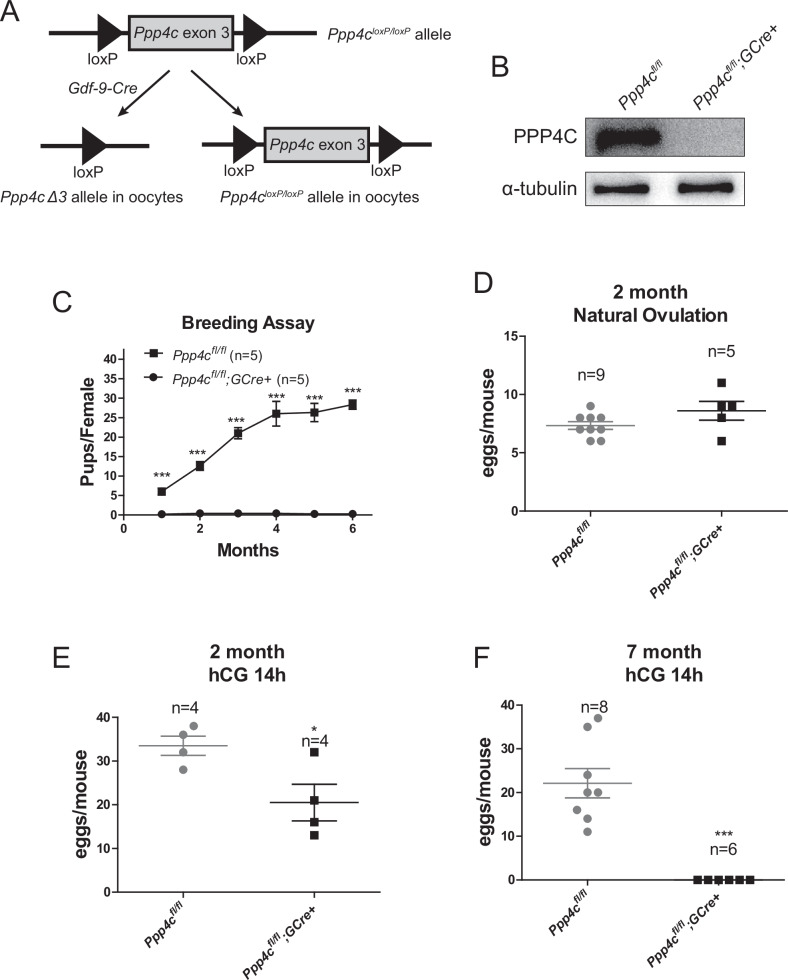
PPP4C is essential for female fertility. **A** Schematic representation of deletion of *Ppp4c* exon 5 and creation of a *Ppp4c* Δ3 allele by *Gdf-9-Cre*-mediated recombination in oocytes. **B** Western blots showing the deletion of *Ppp4c* from mouse oocytes. **C** infertility of the *Ppp4c*^*fl/fl*^*;GCre+* female mice. Data showing the cumulative number of pups per female after continuous breeding for 6 months. Five mice of each genotype were used. **D** Natural ovulation of 2-month-old control and *Ppp4c* knockout mice. Fertilized eggs were collected and counted from females with vaginal plugs after mating. Superovulation of control and *Ppp4c* knockout mice when they were 2 (**E**) and 7 (**F**) months old. MII oocytes were collected and counted 14–16 h after hCG injection. Data are expressed as mean±s.e.m. *P < 0.05 and ***P < 0.001. The total numbers of analyzed mice are indicated (*n*).

To study the functional roles of PPP4 in mammalian oocytes during folliculogenesis, we deleted the *Ppp4c* gene from mouse oocytes by generating mutant mice (referred to as *Ppp4c*^*fl/fl*^*;GCre+* mice), by crossing *Ppp4c*^*fl/fl*^ mice [34] with transgenic mice expressing growth differentiation factor 9 (*Gdf9*) promoter-mediated Cre recombinase (Fig. 1A); this recombinase is active specifically in oocytes during the primordial follicle stage [35]. By immunoblotting analysis, we confirmed successful depletion of PPP4C protein in oocytes from *Ppp4c*^*fl/fl*^*;GCre+* females (Figs. 1B and S8A).

To investigate the effect of oocyte-specific knockout of PPP4C on female fertility, a breeding assay was carried out by mating 6 weeks old *Ppp4c*^*fl/fl*^ or *Ppp4c*^*fl/fl*^*;GCre+* female mice with wild-type C57BL/6J background males of proven fertility for 6 months. We checked daily for counting the cumulative total number of litters and pups per month. As shown in Fig. 1C, *Ppp4c*^*fl/fl*^*;GCre+* female mice were completely infertile in early adulthood. To explore the reason for infertility, natural ovulation without any hormone treatment was assessed firstly when the mice were 2 months old. However, the number of eggs was not significantly different between mutant mice and the control (Fig. 1D, 8.60 ± 0.72 versus 7.33 ± 0.31, *P* > 0.05). Meanwhile, superovulation treated with hormone assay showed that the number of ovulated metaphase II (MII) oocytes in 2- and 7-month-old *Ppp4c*^*fl/fl*^*;GCre+* mice were decreased and disappeared, respectively, compared with the control mice (Fig. 1E, F).

### Depletion of PPP4C impairs genomic integrity in oocytes and early embryos but does not affect oocyte meiotic maturation when the mice are 2 months old

Ovulation disappearance in *Ppp4c*^*fl/fl*^*;GCre+* mice could only explain why they were infertile when they were 7 months old, but could not explain why they were infertile in earlier adulthood. To explore the reason for infertility when the mice are 2 months old, we first compared the oocyte meiotic maturation progression and genomic integrity in mutant mice with the control. The germinal vesicle stage (GV) oocytes were employed for culture in vitro to observe the major events. As shown in Fig. S1, there was no significant difference in PBE (the first polar body extrusion) rates (Fig. S1A, B) and spindle organization (Fig. S1C, D) between the *Ppp4c* knockout and the control groups, which demonstrated that PPP4C has no influence on the oocyte meiotic maturation. Nonetheless, single-cell gel electrophoresis (comet assay) showed that MII oocytes had large amounts of DNA damage (Fig. S1E).

**Fig. 2:**
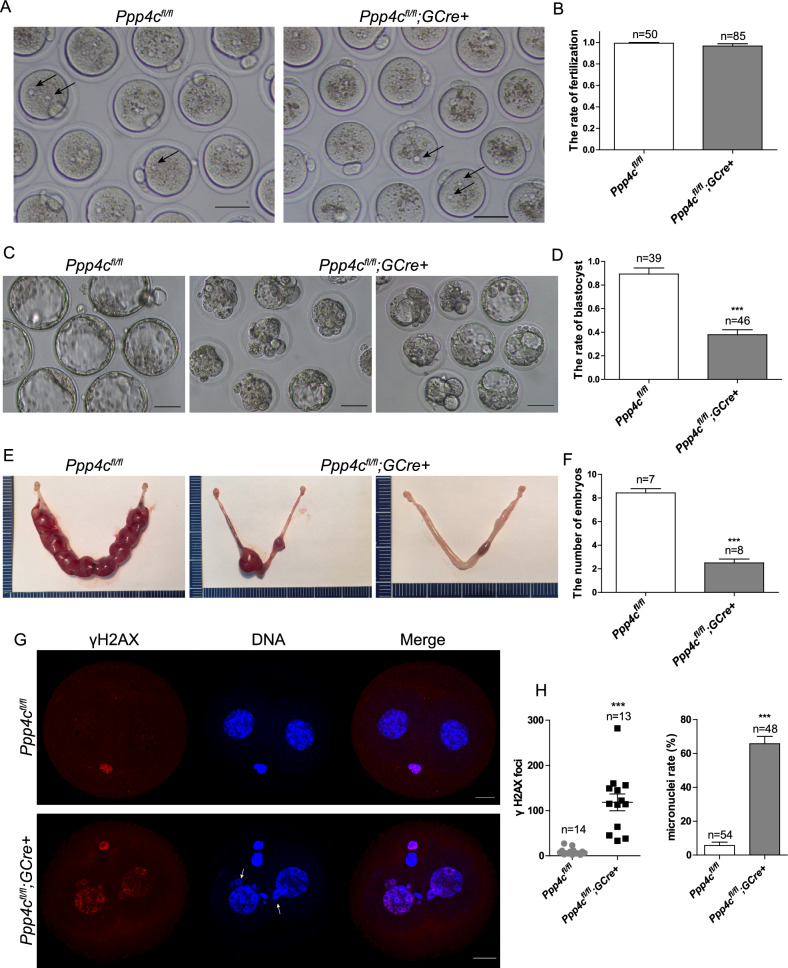
Maternal depletion of PPP4C leads to developmental arrest and accumulated DNA damage in embryos. **A**, **B** *Ppp4c*^*fl/fl*^ and *Ppp4c*^*fl/fl*^*;GCre+* embryos at E0.5 were flushed from the oviducts of mated females with plug. Arrows point to pronuclei. Scale bars: 50 μm. **C**, **D** *Ppp4c*^*fl/fl*^ and *Ppp4c*^*fl/fl*^*;GCre+* embryos at E4.5 cultured in KSOM. Scale bars: 50 μm. **E**, **F** *Ppp4c*^*fl/fl*^ and *Ppp4c*^*fl/fl*^*;GCre+* embryos at E10.5 in uterus. **G**, **H** Two-cell embryos of *Ppp4c*^*fl/fl*^ and *Ppp4c*^*fl/fl*^*;GCre+* females were analyzed by γH2AX and DAPI staining. Arrows in (**G**) point to the micronucleus. Scale bars: 20 μm. Data are presented as mean ± s.e.m. ***P < 0.001. The numbers of analyzed embryos (**B**, **D** and **G**) or mice (**F**) are indicated (*n*). Each experiment was repeated at least 3 times.

The MII oocytes with damaged genome could be successfully fertilized and the second polar bodies were extruded (Fig. 2A, B). Next, the mouse embryos recovered at E0.5 were cultured in vitro to develop into the blastocyst stages. After 4 days culture, only 38.3 ± 3.4% of embryos from *Ppp4c*^*fl/fl*^*;GCre+* mice could form blastocoele, among which many showed abnormalities in morphology (Fig. 2C, D). In vivo, even though a few mutant embryos could implant in uterus, the average number of embryos at E10.5 was significantly lower and a proportion of embryos had been died (Fig. 2E, F). To determine whether genomic integrity of early embryos was impaired, γH2AX was assessed in the G2 phase of two-cell embryos (48 h post-hCG) by staining. As shown in Fig. 2G, H, accumulation of γH2AX and abnormal micronuclei in *Ppp4c*^*fl/fl*^*;GCre+* embryos demonstrated that DNA lesions could not be repaired due to PPP4C deficiency. In sum, unrepaired DNA damage in PPP4C-deficient oocytes and embryos resulted in infertility of mutant mice at earlier adulthood.

### Oocyte-specific deletion of *Ppp4c* causes primordial follicles over-loss followed by premature ovarian failure

**Fig. 3:**
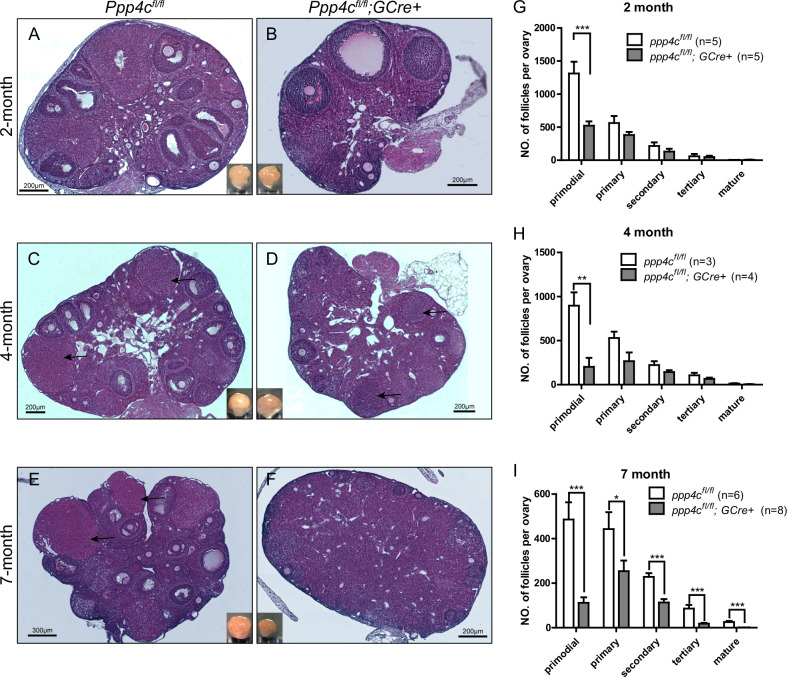
Premature ovarian failure in *Ppp4c*^*fl/fl*^*;GCre+* female mice. **A**–**F** Histology of ovarian sections from 2-, 4- and 7-month-old *Ppp4c*^*fl/fl*^ and *Ppp4c*^*fl/fl*^*;GCre+* females stained with hematoxylin and eosin. The arrows point to corpora lutea. **G**–**I** Numbers of different types of follicles per ovary from 2-, 4- and 7-month-old *Ppp4c*^*fl/fl*^ and *Ppp4c*^*fl/fl*^*;GCre+* females were counted, including numbers of primordial, primary, secondary, tertiary and mature follicles. The experiments were repeated more than 3 times, and for each time and each age, ovaries from one mouse of each genotype were used. Data are presented as mean ± s.e.m. *P < 0.05, **P < 0.01, ***P < 0.001. The numbers of analyzed ovary are indicated (*n*).

Follicular development was quantified to explore the basis for the reduced and disappeared ovulation at 2 and 7 months of age, respectively. At 2 months of age, histological assessment revealed that *Ppp4c*^*fl/fl*^ mice showed normal ovarian morphology characterized by the presence of primordial and activated follicles including primary, secondary, tertiary and mature follicles (Fig. 3A). All of these structures were also found in the *Ppp4c*^*fl/fl*^*;GCre+* ovaries which looked healthy on the whole (Fig. 3B). However, quantitative analysis revealed that the number of primordial follicles in *Ppp4c*^*fl/fl*^*;GCre+* ovaries was markedly reduced as compared to *Ppp4c*^*fl/fl*^ ovaries (Fig. 3G). Activated follicles in *Ppp4c*^*fl/fl*^*;GCre+* ovaries tended to be decreased than that in control ovaries, but the difference in number was not statistically significant (Fig. 3G). At 4 months of age, all types of follicles and corpora lutea (CL) could also be found in both *Ppp4c*^*fl/fl*^ ovaries and *Ppp4c*^*fl/fl*^*;GCre+* ovaries (Fig. 3C, D). It displayed a similar trend at 4 months of age, and primordial follicles in *Ppp4c*^*fl/fl*^*;GCre+* ovaries was markedly reduced and activated follicles tended to be decreased than that in control ovaries, but the difference in number was not statistically significant (Fig. 3H). By 7 months old, primordial and activated follicles in *Ppp4c*^*fl/fl*^*;GCre+* ovaries were both significantly decreased (Fig. 3E, F and I), and CL was rarely found in the ovaries (Fig. 3F), which demonstrated that the ovaries lose the ability of ovulation.

### PPP4C is necessary for formation of primordial follicle pool

**Fig. 4:**
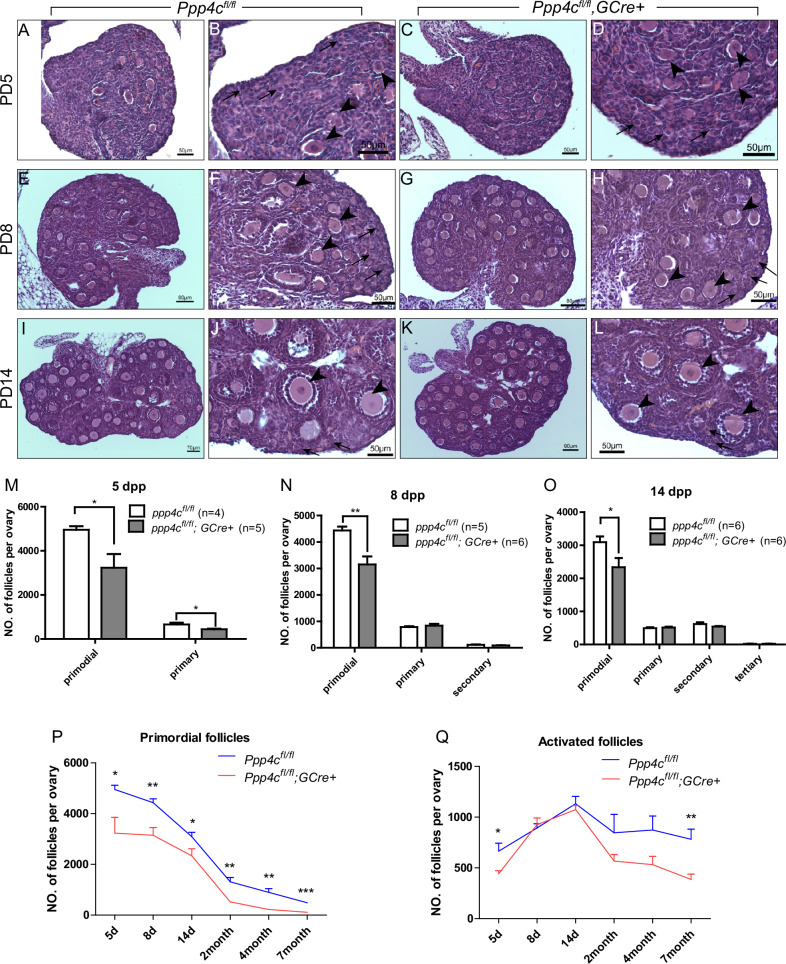
PPP4C is necessary for formation of primordial follicle pool. **A**–**L** Histology of ovarian sections from 5-, 8- and 14-day-old *Ppp4c*^*fl/fl*^ and *Ppp4c*^*fl/fl*^*;GCre+* females stained with hematoxylin and eosin. The arrows point to primordial follicles; the arrowheads point to activated follicles. **M**–**O** Numbers of different types of follicles per ovary from 5-, 8- and 14-day-old *Ppp4c*^*fl/fl*^ and *Ppp4c*^*fl/fl*^*;GCre+* females were counted, including numbers of primordial, primary, secondary and tertiary follicles. The experiments were repeated more than 3 times, and for each time and each age, ovaries from one mouse of each genotype were used. Data are presented as mean ± s.e.m. *P < 0.05, **P < 0.01. The numbers of analyzed ovary are indicated (*n*). Numbers of primordial follicles (**P**) and activated follicles (**Q**) per ovary from 5-day, 8-day, 14-day, 2-month, 4-month and 7-month-old *Ppp4c*^*fl/fl*^ and *Ppp4c*^*fl/fl*^*;GCre+* females were counted. For each time point, at least 3 mice of each genotype were used. Data are presented as mean ± s.e.m. *P < 0.05, **P < 0.01, ***P < 0.001.

To investigate when the primordial follicles start to decline resulting in poor ovarian reserve, further histological analysis of ovaries from 5-, 8- and 14-day-old *Ppp4c*^*fl/fl*^ and *Ppp4c*^*fl/fl*^*;GCre+* females was carried out. We found no apparent morphological difference in postnatal day (PD5) ovaries of *Ppp4c*^*fl/fl*^ and *Ppp4c*^*fl/fl*^*;GCre+* females (Fig. 4A–D), during which the first wave of postnatal follicular development is happening. The ovaries of both genotypes had mostly primordial follicles containing small oocytes surrounded by flattened pregranulosa cells (Fig. 4B, D, arrows) and some primary follicles containing enlarged oocytes (Fig. 4B, D, arrowheads). However, we found that the two types of follicles (primordial and primary) in *Ppp4c*^*fl/fl*^*;GCre+* ovaries were both significantly decreased by quantification analysis (Fig. 4M), of which more than 1000 primordial follicles were over-lost compared with that in control. By PD8 and PD14, the morphology (Fig. 4E–L) and numbers (Fig. 4N, O) of activated follicles in *Ppp4c*^*fl/fl*^*;GCre+* ovaries were similar to those of control, but the primordial follicle pool remained fewer than the *Ppp4c*^*fl/fl*^ ovaries (Fig. 4N, O). Overall, the primordial follicles reduction occurred before PD5 and continued decreasing until exhausting the primordial follicle pool of *Ppp4c*^*fl/fl*^*;GCre+* mice at young adulthood (7 months old) compared to *Ppp4c*^*fl/fl*^ mice (Fig. 4P). The reduction of activated follicles in *Ppp4c*^*fl/fl*^*;GCre+* mice appeared at PD5, but the number of them caught up to the control at PD8 and remained comparable until 7 months old when the primordial follicle pool was exhausted (Fig. 4Q).

### *Ppp4c* deletion results in increased mTOR activation and autophagy flux blockage

**Fig. 5:**
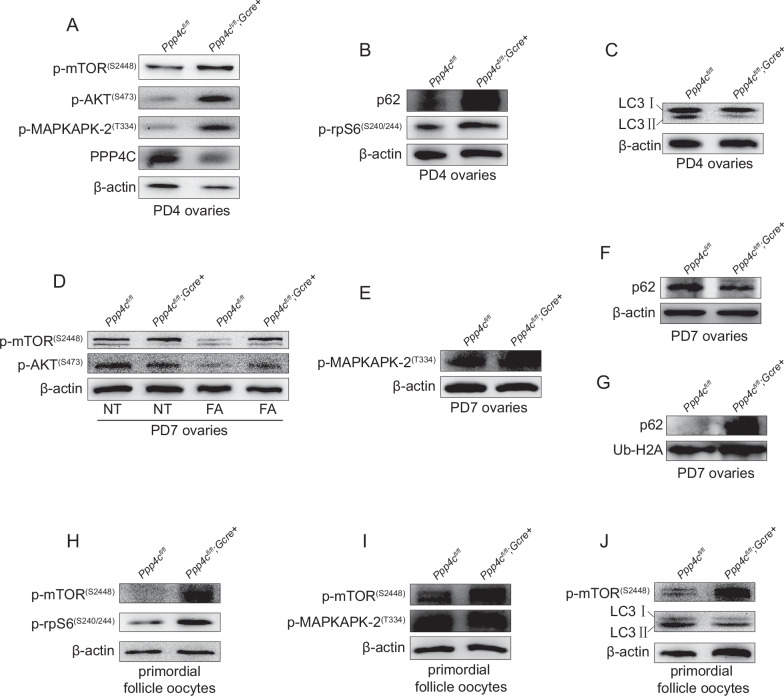
PPP4C deficiency induces mTOR hyperactivation and inhibits p62 degradation. **A**–**C** Signaling studies in *Ppp4c*^*fl/fl*^ and *Ppp4c*^*fl/fl*^*;GCre+* ovaries at PD4, showing levels of p-mTOR^S2448^, p-AKT^S473^, p-MAPKAPK-2^T334^, PPP4C, p-rpS6^S240/244^, P62 and LC3B. Level of β-actin was detected as internal control. **D**, **E** Western blots showing levels of p-mTOR^S2448^, p-AKT^S473^ and p-MAPKAPK-2^T334^ in *Ppp4c*^*fl/fl*^ and *Ppp4c*^*fl/fl*^*;GCre+* ovaries at PD7. Mice were treated with (FA) or without (NT) fasting for 2 days before sacrifice. Level of β-actin was detected as internal control. **F** Western blot analysis of p62 in soluble lysates of PD7 ovaries. Level of β-actin was detected as internal control. **G** Western blot analysis of p62 in insoluble lysates of PD7 ovaries. Level of Ub-H2A was detected as internal control. **H**–**J** Signaling studies in *Ppp4c*^*fl/fl*^ and *Ppp4c*^*fl/fl*^*;GCre+* primordial follicle oocytes at PD7, showing levels of p-mTOR^S2448^, p-rpS6^S240/244^, p-MAPKAPK-2^T334^ and LC3B. Level of β-actin was detected as internal control. The ovary lysates or oocytes were collected at least from three mice of each genotype. Each experiment was repeated at least 3 times.

We found that depletion of PPP4C impairs genomic integrity in MII oocytes (Fig. S1E), so we tried to explore if the DNA lesions existed in primordial follicle oocytes, causing eventual oocyte elimination. Westen blot analysis showed that PPP4C had been knocked out in oocytes at PD4 (Fig. 5A), PD8 and PD14 (Figs. S2A and S8B). However, there was no significant difference of DNA lesions and apoptosis between *Ppp4c*^*fl/fl*^ and *Ppp4c*^*fl/fl*^*;GCre+* ovaries at PD8 (Fig. S2B, C), which demonstrated that depletion of PPP4C do not induce apoptosis in primordial follicle oocytes. Accumulating literatures have revealed that PI3K/AKT signaling plays a vital role in regulating the survival of primordial follicles [25, 36]. To elucidate the molecular mechanism underlying the primordial follicle over-loss in *Ppp4c*^*fl/fl*^*;GCre+* ovaries, we studied AKT signaling in ovaries of PD4 *Ppp4c*^*fl/fl*^ and *Ppp4c*^*fl/fl*^*;GCre+* mice. We found that the level of phosphor-AKT (p-AKT, Ser^473^) was elevated in *Ppp4c*^*fl/fl*^*;GCre+* ovaries (Figs. 5A, S3A and D). Meanwhile, we also studied the upstream activator and substate of AKT, MAPKAPK-2 [37] and mTOR [38], respectively. As shown in Fig. 5A, MAPKAPK-2 and mTOR maintained hyperphosphorylation in *Ppp4c*^*fl/fl*^*;GCre+* ovaries than the control. In addition, enhanced mTOR signaling facilitated the phosphorylation of rpS6 (Fig. 5B), a downstream effector of mTOR [36]. As a result of the inverse relationship between mTOR and autophagy, we speculated that the PPP4C deficiency might cause autophagy defects. To confirm the hypothesis, we analyzed the amount of autophagosome membrane-associated form LC3B II and autophagic substrate p62 in ovaries at PD4 and found that LC3B II was reduced and p62 was accumulated in *Ppp4c*-deficient ovaries (Figs. 5B, C, S3B–D and E).

Starvation could increase autophagy in mouse oocytes, which plays a reverse role contrast to AKT-mTOR on autophagy [39]. At PD7, suffering fasting for 2 days, the level of phosphor-mTOR (p-mTOR, Ser^2448^) in *Ppp4c*^*fl/fl*^*;GCre+* ovaries was still higher than in *Ppp4c*^*fl/fl*^ ovaries (Figs. 5D, and S4A, C), which indicated that PPP4C-deficient ovaries were less sensitive to starvation. However, AKT remained lower level of phosphorylation at PD7 in *Ppp4c*^*fl/fl*^*;GCre+* ovaries than the control and could be dephosphorylated when mice were treated with fasting (Figs. 5D and S4D). In addition, PPP4C deficiency did not affect the phosphorylation of MAPKAPK-2 at PD7 (Figs. 5E andS4B, E). As an autophagy adaptor, SQSTM1/p62 (p62) is able to recruit polyubiquitinated cargos into autophagosomes via its LC3-interacting region domain and eventually degraded by autophagy [40, 41]. On the other hand, inhibition of autophagy activity can lead to the accumulation of p62 associated with ubiquitinated proteins, resulting in high cytoplasmic levels of oligomerized insoluble p62 and forming p62-positive inclusions [42]. Although the level of soluble p62 was decreased in *Ppp4c*^*fl/fl*^*;GCre+* ovaries at PD7 (Figs. 5F and S4F, H), the level of insoluble p62 was significantly elevated in *Ppp4c*^*fl/fl*^*;GCre+* ovaries (Figs. 5G and S4G, I). These results indicated that the homeostatic level of p62 had been broken due to PPP4C deficiency.

To further accurately verify our speculation, we studied mTOR signaling in primordial follicle oocytes isolated from ovaries of PD7 *Ppp4c*^*fl/fl*^ and *Ppp4c*^*fl/fl*^*;GCre+* mice. We found that the level of phosphor-mTOR and phosphor-rpS6 was elevated in *Ppp4c*^*fl/fl*^*;GCre+* primordial follicle oocytes (Figs. 5H and S5A, C), but the level of phosphor-MAPKAPK-2 was not affected (Figs. 5I and S5B, C). Meanwhile, we found that LC3B II was reduced in *Ppp4c*-deficient primordial follicle oocytes (Figs. 5J and S5D, E). However, *Ppp4c* deletion did not influence the level of phospho-mTOR and phospho-rpS6 in GV oocytes (Fig. S8C), which means that PPP4C might regulate phosphorylation of mTOR and autophagy only in primordial follicle oocytes.

### *Ppp4c* deletion results in p62 accumulation and inclusion formation in oocytes

**Fig. 6:**
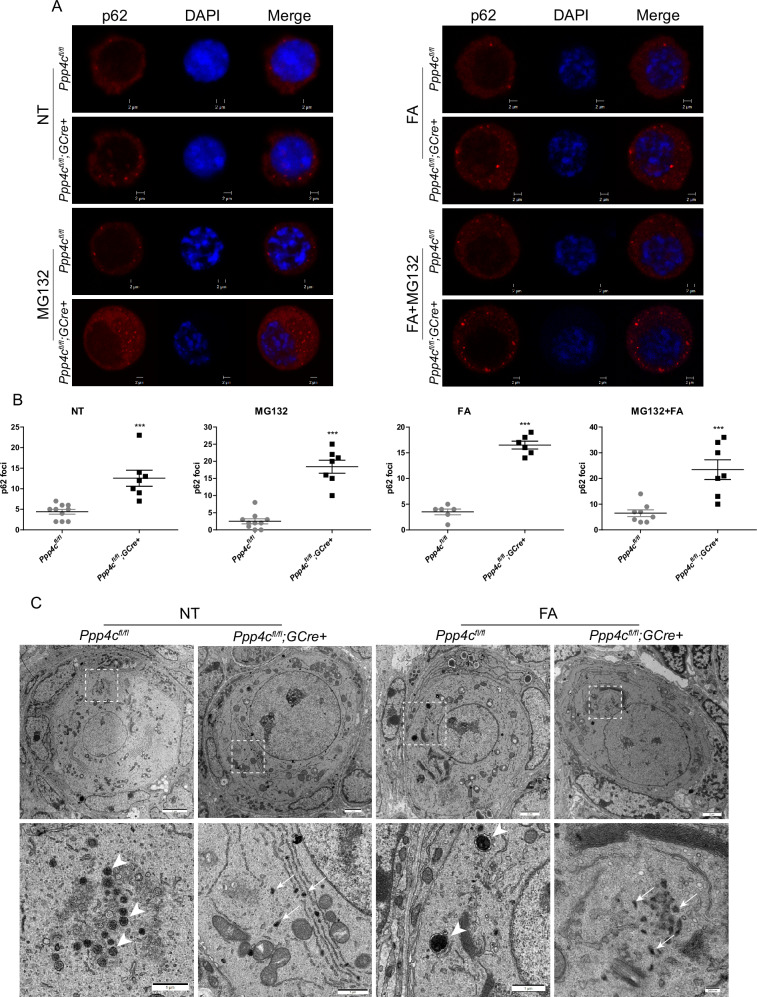
PPP4C deficiency induces p62 accumulation and inclusion formation in oocytes. **A** Representative images of p62 foci in untreated (NT), MG132, fasting (FA) or MG132 and fasting (MG132 + FA) treated primordial follicle oocytes of *Ppp4c*^*fl/fl*^ and *Ppp4c*^*fl/fl*^*;GCre+* females at PD7. Scale bars: 2 μm. **B** Quantification of p62 foci in A was analyzed with ImageJ software. **C** Electron micrograph of primordial follicle oocytes of *Ppp4c*^*fl/fl*^ and *Ppp4c*^*fl/fl*^*;GCre+* females at PD7. Mice were treated with (FA) or without (NT) fasting for 2 days before sacrifice. Note that some electron dense materials surrounded by membranes form autophagosome (arrows) in *Ppp4c*^*fl/fl*^ oocytes, which could be degraded by lysosomal hydrolysis. Instead, the electron dense materials could not be surrounded by membranes in *Ppp4c*^*fl/fl*^*;GCre+* oocytes. Along with the accumulation, aggresomes or inclusions were formed (arrowheads). Data are presented as mean ± s.e.m. ***P < 0.001. Each experiment was repeated at least 3 times.

The results of Western blot analysis gave us a hint: autophagy was inhibited in PPP4C-deficient primordial follicle oocytes. To confirm the hypothesis, we analyzed the p62 foci, whose total cellular expression level inversely correlated with autophagic activity [43], in primordial follicle oocytes. Immunofluorescence microscopy showed that the *Ppp4c*-deficient oocytes contained abundant p62-positive foci in the cytoplasm (Fig. 6A, B), no matter treated with or without autophagic activators (MG132, fasting) [44], which demonstrated that the turnover of p62 had been impaired. Although MG132 is an autophagy activator, it is a proteasome inhibitor. Therefore, MG132 treatment induces a decrease of p62 foci in wild-type mice but an increase of p62 foci in PPP4C-depleted mice due to both lack of ubiquitin-proteasome system and autophagy in *Ppp4c*-deficient oocytes. On the other hand, p62 is a component of inclusions or aggresomes when autophagy is deficient [40]. The anomalous accumulation of p62 means more inclusions or aggresomes are present in cytoplasm and the degradation ability of autophagy is lower. As shown in Fig. 6C, there are a lot of protein aggregations (electron-dense materials), which are amorphous without being surrounded by membranes, in the cytoplasm of *Ppp4c*-deficient primordial follicle oocytes. After fasting treatment, we could find that the amorphous small aggregates were transported near a centriole, a common feature of aggresome or inclusion [45]. Instead, the materials surrounded by membranes form autophagosome in *Ppp4c*^*fl/fl*^ oocytes, which could eventually be degraded by lysosomal hydrolysis (Fig. 6C).

### Autophagy induction is disrupted in the primordial follicle oocytes of *Ppp4c*^*fl/fl*^*;GCre+* mice

**Fig. 7:**
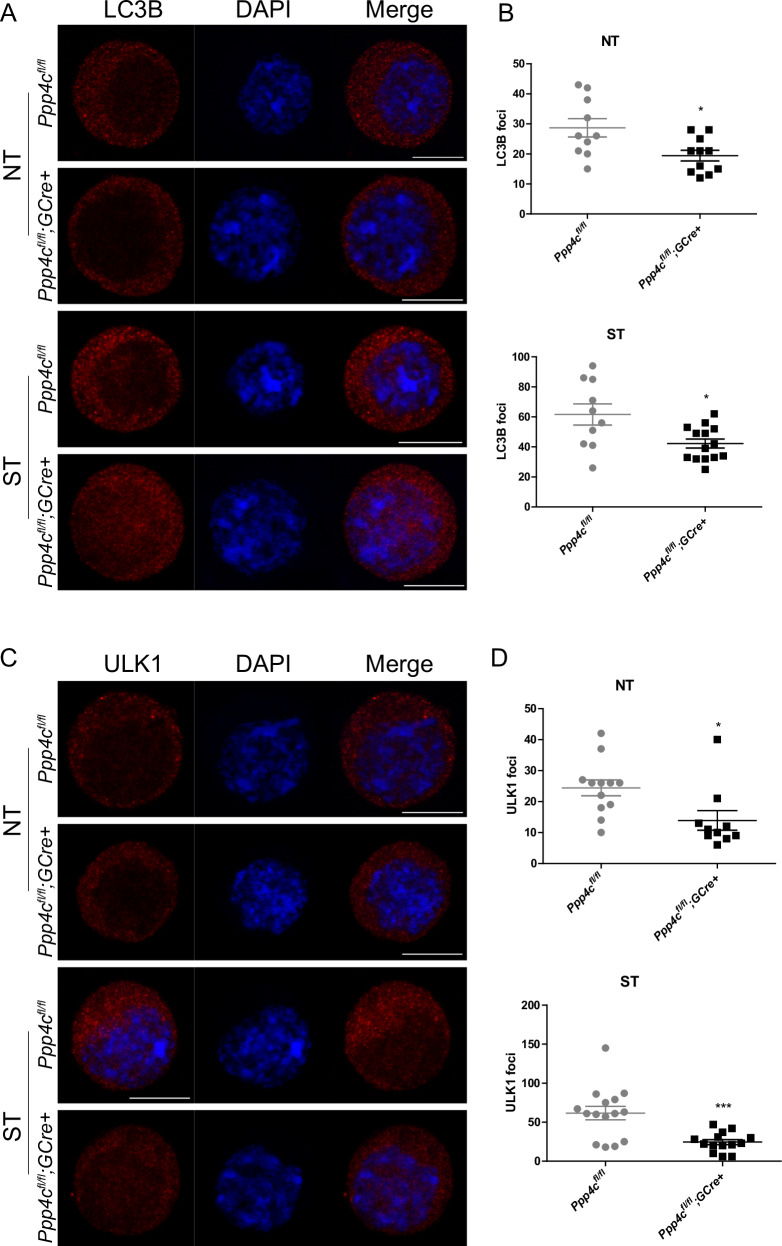
PPP4C deficiency inhibits autophagy induction. **A** Representative images of LC3B foci in untreated (NT) or starvation (ST) treated primordial follicle oocytes of *Ppp4c*^*fl/fl*^ and *Ppp4c*^*fl/fl*^*;GCre+* females at PD5-7. **B** Quantification of LC3B foci in (**A**) was analyzed with ImageJ software. **C** Representative images of ULK1 foci in untreated (NT) or starvation (ST) treated primordial follicle oocytes of *Ppp4c*^*fl/fl*^ and *Ppp4c*^*fl/fl*^*;GCre+* females at PD5-7. **D** Quantification of ULK1 foci in (**C**) was analyzed with ImageJ software. Data are presented as mean ± s.e.m. *P < 0.05, ***P < 0.001. Scale bars: 10 μm. Each experiment was repeated at least 3 times.

The inhibitory function of mTOR in autophagy induction is well established [18, 46, 47]. Based on the above results, we speculated that the autophagy initiation might be blocked in the in *Ppp4c*-deficient primordial follicle oocytes. To verify this hypothesis, we estimated the amount of the LC3B in PD5-7 primordial follicle oocytes between *Ppp4c*^*fl/fl*^ and *Ppp4c*^*fl/fl*^*;GCre+* mice. Immunofluorescence microscopy showed that the *Ppp4c*-deficient primordial follicle oocytes contained fewer LC3B foci than control (Figs. 7A, B and S6A, B), which demonstrated that the formation of autophagosomes was suppressed by deletion of PPP4C.

ULK1, a key regulator in autophagy initiation, is a direct substrate of mTOR at multiple sites including S637 and S757 in murine ULK1 and sustaining phosphorylation at S637 could diminish its translocation to punctate structure [47]. According to this characteristic, we observed the translocation of ULK1. As shown in Fig. 7C, D, the punctate structure of ULK1 was decreased in *Ppp4c*-deficient primordial follicle oocytes, which demonstrated that the initiation of autophagy was altered.

### The blocked autophagic flux in *Ppp4c*-deficient primordial follicle oocytes can be restored by inhibiting mTOR

**Fig. 8:**
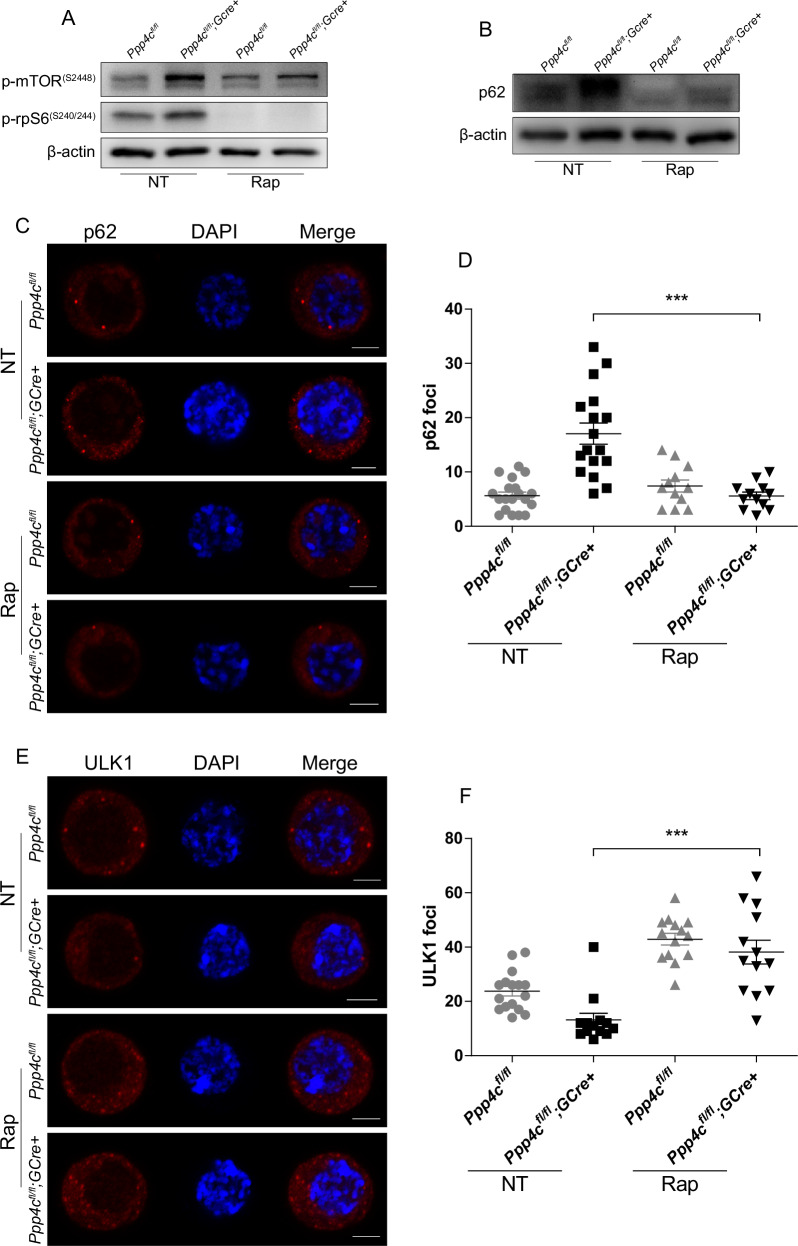
The blocked autophagic flux in *Ppp4c*-deficient primordial follicle oocytes could be restored by inhibiting mTOR. **A**, **B** Signaling studies in *Ppp4c*^*fl/fl*^ and *Ppp4c*^*fl/fl*^*;GCre+* ovaries at PD4, showing levels of p-mTOR^S2448^, p-rpS6^S240/244^ and P62. Level of β-actin was detected as internal control. Ovaries were treated with 300 nm rapamycin (Rap) or not (NT) for 6 h before adding lysis buffer. **C** Representative images of p62 foci in untreated (NT) or rapamycin (Rap) treated primordial follicle oocytes of *Ppp4c*^*fl/fl*^ and *Ppp4c*^*fl/fl*^*;GCre+* females at PD5. Scale bars: 5 μm. **D** Quantification of p62 foci in (**C**) was analyzed with ImageJ software. **E** Representative images of ULK1 foci in untreated (NT) or rapamycin (Rap) treated primordial follicle oocytes of *Ppp4c*^*fl/fl*^ and *Ppp4c*^*fl/fl*^*;GCre+* females at PD5. Scale bars: 5 μm. **F** Quantification of ULK1 foci in E was analyzed with ImageJ software. Data are presented as mean ± s.e.m. ***P < 0.001. Each experiment was repeated at least 3 times.

To explore the relationship between mTOR and autophagy in primordial follicle oocytes, a rescue experiment was performed. Two ovaries from one 4 days old mouse were respectively treated with or without 300 nM rapamycin (an inhibitor of mTOR) for 6 h. Although the phosphorylation on Ser2448 of mTOR could not be blocked by rapamycin [48], the dephosphorylation of downstream effector rpS6 demonstrated that the kinase activity of mTOR had been inhibited (Figs. 8A and S7A, C and D). Meanwhile, p62 accumulation in *Ppp4c*-deficient ovaries could be reversed by successful inhibition of mTOR (Figs. 8B and S7B, E). Immunofluorescence microscopy also showed that accumulated p62 foci in *Ppp4c*-deficient primordial follicle oocytes could be erased by inhibiting mTOR in oocytes by treatment with 1 μM rapamycin for 1 h (Fig. 8C, D). We also analyzed the substrate of mTOR, as shown in Fig. 8E, F, ULK1 foci were increased in *Ppp4c*-deficient primordial follicle oocytes when mTOR was inhibited, which means the blocked autophagic flux in *Ppp4c*-deficient primordial follicle oocytes have been restored by inhibiting mTOR.

### Abnormal primordial follicle oocytes are erased by pregranulosa cells in the manner of lysosome invading

**Fig. 9:**
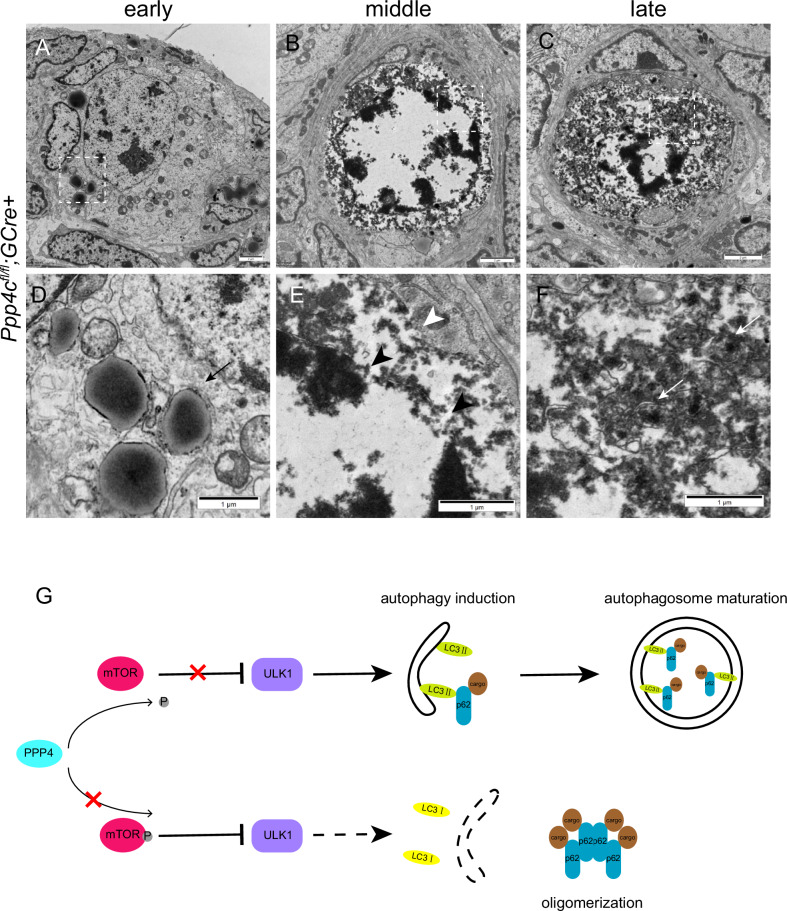
Abnormal primordial follicle oocytes are erased by pregranulosa cells in *Ppp4c*^*fl/fl*^*;GCre+* ovary. Electron micrographs show different stages of oocyte degradation process. **A**, **D** Early stage: lysosome invades from pregranulosa cell to primordial follicle oocyte. Black arrow in (**D**) points to the broken membrane. **B**, **E** Middle stage: cytoplasm and nuclear condensation along with nuclear envelope breakdown (black arrowheads). Parts of the cell membrane of the surrounding pregranulosa cells are lacking (white arrowheads). **C**, **F** Late stage: formation of abundant autophagolysosomes (white arrows). Panels (**D**), (**E**) and (**F**) are magnified images of rectangular areas marked with a dotted line in panels (**A**), (**B**) and (**C**), respectively. **G** A model for PPP4 in regulating the formation of autophagosome. Top: In the presence of PPP4, phosphorylation of mTOR is decreased, and subsequently ULK1 induces autophagy. The damaged cargos are sequestered in autophagy by p62-LC3II interaction. Bottom: when PPP4 is deficient, mTOR is active and ULK1 is inhibited. Insufficient autophagosome causes the accumulation of p62, resulting in high cytoplasmic levels of oligomerized p62.

Even though autophagy process was inhibited in *Ppp4c*-deficient primordial follicle oocytes, the mechanism of oocyte death was not understood. To further unravel this veil, we observed the process of primordial follicle oocyte death by electron microscopy. Result analysis demonstrated a sequence of changes that occurred in abnormal primordial follicle oocytes of *Ppp4c*^*fl/fl*^*;GCre+* females. Figure 9A, D shows the early stage of the primordial oocyte entered cell death. Nucleus began increasing density and lysosome invaded from the broken membrane of pregranulosa cell to primordial follicle oocyte, which differs from the normal oocyte in Fig. 6C. At the middle stage, the primordial follicle oocyte consisted of a loop of strongly condensed globular chromosomes surrounded by the condensed cytoplasm (Fig. 9B). The cell membrane of the pregranulosa cell that encompassed the oocyte was lacking in parts (Fig. 9E). Nuclear envelope of oocyte was also broken down (Fig. 9E). At the late stage, abundant autophagolysosomes were formed in the degenerating cell (Fig. 9C, F).

In summary, this study demonstrates the functional importance of PPP4 in regulating autophagy induction by inhibiting mTOR. Subsequently, the ULK1 autophagy initiation complex is activated and assembled, following autophagy initiation and autophagosome maturation. Finally, the damaged organelles or misfolded proteins are sequestered in autophagosomes (Fig. 9G).

## Discussion

In the mammalian ovary, the pool of primordial follicles serves as the source of developing follicles and fertilizable ova for the entire duration of reproductive life. In this study, by using a mouse model with oocyte-specific deletion of *Ppp4c*, we found that PPP4C in oocytes plays an essential role in preserving the normal reproductive ability in females. On one hand, PPP4C safeguards MII oocytes and early embryo genomic integrity. Our recent study showed that *Zp3-Cre* mediated PPP4C depletion in growing oocytes impaired homologous recombination DNA repair by phosphorylating PLK1 during early embryo development [34]. On the other hand, the current study showed that deletion of *Ppp4c* in mouse primordial follicle oocytes by *Gdf9-Cre* results in accelerated ovarian reserve loss, by causing suppressed autophagy in primordial oocytes. This phenotype was not found in *Zp3-Cre* mediated PPP4C depletion females. Studies have reported that *Gdf9* is expressed in primordial follicle oocytes at as early as birth [49], while the synthesis of ZP3 starts in primary follicles from PD5 [35]. The different phenotypes between the two knockout mouse models not only reflect differences between *Zp3-Cre* and *Gdf9-Cre*-caused phenotypes-, but also indicated that PPP4 is essential for primordial follicle pool maintenance. Based on the evidence we provided, the primordial follicles reduction occurred before PD5 and continued decrease until exhausting the primordial follicle pool in *Ppp4c*^*fl/fl*^*;GCre+* mice.

Autophagy, a process of self-eating of cellular materials, is requisite to reprocess the damaged cell organelles or materials to procure new building blocks for maintaining cellular homeostasis [14]. Numerous studies revealed autophagy is necessary for maintaining the development and survival of oocytes [9, 14, 50]. We found that the over-loss of primordial follicles in *Ppp4c*^*fl/fl*^*;GCre+* ovaries is due to elevated mTOR activity, which inhibits the autophagy and enhances the activation of rpS6 that promotes protein translation in oocytes. Thus, we speculate the homeostasis of protein metabolism in *Ppp4c*^*fl/fl*^*;GCre+* primordial follicle oocytes might have been impaired: the misfold protein could not be degraded which may result in lacking raw materials to synthesize new protein due to autophagic flux blockage. AKT/TOR is the classical signaling pathway that controls autophagy in yeast and higher eukaryotes. In yeast, TOR negatively controls autophagy via inhibition of the protein kinase ATG1 that mediates an early activation step in the autophagic process [38, 51]. Similar to yeast TOR, *Drosophila* dTOR suppresses autophagy by a mechanism that involves ATG1 [39]. Although mTOR is a major gatekeeper and inhibitor of autophagy in mammalian species, the molecular connection between mTOR and the machinery required for autophagy is not yet firmly established. Our data show that oocyte PPP4C functions as a suppressor of mTOR signaling and facilitates the initiation of autophagy by activating the ULK1 complex. The activation of AKT, the upstream regulators of mTOR, and MAPKAPK-2, the upstream activator of AKT, were also dramatically elevated in PD4 *Ppp4c*^*fl/fl*^*;GCre+* ovaries, which suggests PPP4 might participate the regulation. However, the activation of AKT was reduced at PD7 *Ppp4c*^*fl/fl*^*;GCre+* ovaries. This dynamic change of AKT may involve in the negative feedback loop, in which activation of mTOR signaling strongly represses PI3K/AKT signaling upstream [52]. On the other hand, it was reported that PTEN, a phosphatase, is major negative regulator of PI3K/AKT signaling [1], which may overlap with PPP4 on the regulation. It is well known that AMPK is a negative regulator of mTOR. Although PPP4 might regulate phosphorylation of AMPK(Thr172) in stromal-vascular fractions cells [53], there are many other protein phosphatases that could dephosphorylate AMPK at this site, for example, PPP2C, PPP2A, PPP6, and PTEN [54–57]. Even though the phosphorylation of AMPK(Thr172) was increased in oocytes, the activity of mTOR was not suppressed [29]. Thus, we are not sure whether the activity of AMPK is altered in our mice model. As for MAPKAPK-2, there may be other unknown signaling pathways regulating its activity in mouse oocytes after PD5, a period that follicles begin to be activated, so PPP4C deficiency did not affect its phosphorylation since then.

Although apoptosis has been suggested as the mechanism underlying primordial follicle oocyte death in postnatal ovary by culture in vitro [7, 58], it has been difficult to detect apoptosis in mouse primordial follicles in vivo using normal apoptotic makers, such as by TUNEL assay, activation of caspase 3, cleavage of PARP or morphological signs of apoptosis [3, 11, 13, 59]. Similarly, we did not find any significant difference of apoptosis between *Ppp4c*^*fl/fl*^*;GCre+* primordial follicle oocytes and the control at PD8, which might mean primordial follicle loss in PPP4C deficient ovaries is not due to apoptotic pathways. These controversies about the mechanisms of primordial follicle loss suggest that there are many differences between physiological oocyte cell death and apoptosis, suggesting that oocyte death should be assigned to a different class of cell death other than apoptosis. In fact, it had been reported that a new death mode of postmitotic oocyte differs from the known mechanisms of cell death (apoptosis, necrosis) two decades ago [12]. The new mode of death involved chromosome condensation without fragmentation, an increased density of cytoplasmic constituents, and the broken cell membrane. Finally, the germ cell was continuous with the cytoplasm of the surrounding pregranulosa cells and became subject to a heterophagic process within the supporting cells. Although it is not exactly the same, we find the similar death mode of primordial follicle oocytes existing in *Ppp4c*^*fl/fl*^*;GCre+* ovaries, which revealed that the death mode might be induced by the impairment of autophagy.

The PPP4 holoenzyme includes several multimeric complexes, which consist of a common catalytic subunit, PPP4C, and different regulatory subunits [30, 60]. Different PPP4 complexes have different cellular functions and subcellular localization. For example, PPP4R3β and PPP4R2 are necessary for DNA damage response, but PPP4R3β regulates the nonhomologous end-joining (NHEJ)-mediated repair [32] while PPP4R2 regulates the homologous recombination (HR)-mediated repair [31]. At present, it is not clear which holoenzymes are present in the oocytes. PPP4C also has different cellular functions in different cells. PPP4C regulates microtubule organization through its targets CDK1 and NDEL1 in mouse embryonic fibroblast (MEF) cells [61], while it also facilitates HR-mediated DNA repair by dephosphorylating PLK1 during early embryo development as we recently revealed [34]. Here, we provide evidence that PPP4C acts as critical regulator of autophagy in primordial follicles by negatively regulating mTOR activity. Further studies are needed to explore which regulatory subunit participates in this process so that it is better to provide valuable information for the design of therapeutics for POI.

## Materials and Methods

### Mice

Mice lacking *Ppp4c* in oocytes (referred to as *Ppp4c*^*fl/fl*^*;GCre*+) were generated by crossing *Ppp4c*^*fl/fl*^ mice [34] with *Gdf9-Cre* mice. Both transgenic mouse lines have C57BL/6J genomic background. The mice were housed under controlled environmental conditions with free access to water and food. Light was provided between 08:00 and 20:00. Animal experimental procedures in our study were approved by the Animal Research Committee of the Institute of Zoology, Chinese Academy of Sciences.

### Antibodies and reagents

Commercial antibodies rabbit anti-PPP4C (#ab16475) and mouse anti-MVH (#ab27591) were purchased from Abcam. Mouse anti-α-tubulin (#DM1A) was purchased from Sigma-Aldrich. Mouse anti-γH2AX (#80312), rabbit anti-Caspase-3 (#9579), rabbit anti-p-AKT^S473^ (#4060), rabbit anti-p-mTOR^S2448^ (#5536), rabbit anti-p-MAPKAPK-2^T334^ (#3007), rabbit anti-p-S6K ^T389^ (#9234), rabbit anti-p-rpS6^S240/244^ (#5364), rabbit anti-GAPDH (#5174), mouse anti-β-actin (#3700) and rabbit anti-α-tubulin (#2144) were purchased from Cell Signaling Technology, Inc. Rabbit anti-p62 (#nbp1-48320) was purchased from Novus. Rabbit anti-LC3B (T55992F) and Rabbit anti-ULK1 (T56902F) were gifted from Abmart. Autophagy activator (MG-132) and rapamycin (HY-10219) were purchased from Medchemexpress. Secondary antibodies used for Western blot and immunofluorescence staining were purchased from ZhongShan Golden Bridge Biotechnology Co., LTD (Beijing) and Thermo Fisher Scientific, Inc, respectively.

### Breeding assay

In breeding assays, *Ppp4c*^*fl/fl*^ and *Ppp4c*^*fl/fl*^*;GCre+* genotype female mice with sexual maturity were continually mated to wild C57BL/6J background male mice with known fertility for 6 months. At least 4 mice of each genotype were used. Cages were checked daily for counting the number of litters and pups.

### Natural ovulation and superovulation examination

For the natural ovulation assay, ~2 months old female mice were mated with fertile males overnight. Successful mating was confirmed by the presence of vaginal plugs. Fertilized eggs were harvested from oviducts, counted, and analyzed after removal of the cumulus mass by treatment with 3 mg/ml hyaluronidase (Sigma-Aldrich) in M2 medium (Sigma-Aldrich).

For superovulation, female mice were injected with 10 IU of PMSG followed by 10 IU of hCG after 48 h to promote ovulation. After an additional 14–16 h, oocyte/cumulus masses were surgically removed from oviducts and the numbers of MII oocytes were counted and analyzed after removing the surrounding cumulus cells.

### Culture and collection of mouse oocytes and early embryos

To harvest oocytes or embryos, females were intraperitoneally injected with 10 IU of PMSG followed by 10 IU of hCG after 48 h to promote ovulation. To harvest embryos, the female mice were mated with wild-type fertile males overnight. Oocytes or embryos in vivo were manipulated in M2 at the indicated time points after hCG injection: MII oocytes, 14–16 h; zygotes, 22–30 h; 2-cell embryos, 48 h. GV oocytes were obtained 48 h after PMSG injection. The GV stage oocytes were isolated from ovaries and cultured in M2 medium under paraffin oil at 37 °C, 5% CO_2_ in air, while zygotes were cultured in KSOM medium. Oocytes and zygotes were collected at specific times of culture for immunofluorescence staining or Western blotting.

To harvest primordial follicle oocytes, mice were sacrificed by decapitation, and the ovaries were dissected free of fat and connective tissue using a microscope. Then, the ovaries were placed in 1×PBS buffer with frequent pipetting and the primordial follicle oocytes at the surface of ovary were isolated. Oocytes were collected by centrifugation.

### Comet assay of oocyte chromosome integrity

Oocytes harvested from ~2 months old mice were imposed comet assay as previously described [62] with slight changes. In brief, oocytes were blow carefully to 0.5% LMP (Low-melting-point) agarose layer that was covered by the normal melting agarose on a dish. The dish was incubated at 4 °C for 5 min and then lowered into ice-cold freshly made lysing solution for 1 h. The dish was washed with distilled water for 3 times and then the DNA was unwound with alkaline buffer for 20 min on ice. Electrophorese at 25 V for 20 min. Finally, the cells on the dish were stained with EB (20 μg/ml) for 15 min and then examined with a fluorescence microscope (Leica, Germany).

### Histological analysis and quantification of ovarian follicles

Ovaries used for histological analysis were collected from adult female mice. They were fixed in 4% paraformaldehyde (pH 7.5) overnight at 4 °C, dehydrated, and embedded in paraffin. Paraffin-embedded ovaries were sectioned at a thickness of 8 μm for hematoxylin and eosin (H&E) staining. One or both ovaries from more than three mice of each genotype were used for the analysis.

Quantification of ovarian follicles was performed as previously described [63]. Briefly, to count the numbers of follicles, paraffin-embedded ovaries were serially sectioned at 8 μm thickness and every fifth section was mounted on slides. Then these sections were stained with hematoxylin and eosin for morphological analysis. Ovarian follicles at different developmental stages, including primordial (type 1 and type 2), primary (type 3a and type 3b), secondary (type 4, type 5a and type 5b), tertiary (type 6 and type 7), and mature (type8) follicles were counted in collected sections of an ovary, based on the well-accepted standards established by Peterson and Peters [64]. In each section, only those follicles in which the nucleus of the oocyte was clearly visible were scored and the cumulative follicle counts were multiplied by a correction factor of 5 to represent the estimated number of total follicles in an ovary.

### MG-132, starvation, fasting and rapamycin treatment

For in vitro treatment of MG-132, collected oocytes were treated with MG-132 (10 μg/ml) for 12 h in Dulbecco’s modified Eagle’s medium-F12 (DMEM/F12; Hyclone) supplemented with 10% fetal bovine serum (FBS) and then fixed in 4% paraformaldehyde in PBS for immunofluorescence staining.

For starvation experiments, primordial follicle oocytes were collected and incubated in 1×HBSS for 1 h and then fixed in 4% paraformaldehyde in PBS for immunofluorescence staining.

For fasting protocols, pups were separated from mother at PD5 for 48 h. Then the ovaries were collected and the oocytes were isolated for immunofluorescence staining or Western blotting.

For both treatment of MG-132 and fasting, pups were separated from mother at PD5 for 36 h. Then the oocytes were collected and treated with MG-132 (10 μg/ml) for 12 h in DMEM/F12 medium without FBS followed by fixed in 4% paraformaldehyde in PBS for immunofluorescence staining.

For in vitro treatment of rapamycin, ovaries collected from 4 days old mice were treated with rapamycin (300 nM) for 6 h in culture medium that consisted of DMEM/F12 + α-minimal essential medium (α-MEM) (1:1; Hyclone) supplemented with 10% FBS, 0.23 mM sodium pyruvate, insulin-transferrin-selenium media supplement (ITS-mix, Beyotime). Primordial follicle oocytes collected from 5 days old mice were treated with rapamycin (1 M) for 1 h in above-mentioned culture medium.

### Immunofluorescence and TUNEL assay

Oocytes and embryos were fixed in 4% paraformaldehyde in PBS for 30 min at room temperature followed by permeabilization in membrane permeabilization solution (0.5% Triton X-100) and then blocked in PBS containing 1 mg/ml BSA for 1 h at room temperature. After blocking, the oocytes/embryos were incubated overnight at 4 °C with the antibodies described above at appropriate dilutions. Subsequently, the oocytes/embryos were incubated for 1 h with specific fluorescent secondary antibodies at room temperature after washing three times with washing buffer. Finally, the oocytes/embryos were washed three times again followed by incubation with DAPI for 15 min, and then mounted on glass slides and examined with a laser scanning confocal microscope (Zeiss LSM 880 META, Germany). Z-series images were obtained to cover the maximal radius of individual nuclei and merged for each cell. For p62 foci analysis, each picture was processed with Enhanced Contrast using ImageJ software. The foci in each picture were counted with Find Maxima by altering Noise Tolerance.

For ovaries, paraffin-embedded ovarian tissue sections were deparaffinized, immersed in retrieval solution (10 mM sodium citrate), heated in an autoclave, blocked with 10% normal goat serum, and then incubated overnight with primary antibodies. Localization of primary antibody was performed by incubation of the sections with the corresponding secondary antibodies at 1:500 dilution for 1 h at room temperature. Finally, nuclei were stained with DAPI.

Analysis of apoptosis in ovarian follicles was carried out by TUNEL assay using the ApopTag Plus in situ apoptosis detection kit (Chemicon International, Temecula, CA, USA).

### Preparation of soluble and insoluble fractions and Western immunoblotting

Ovaries were lysed on ice using a homogenizer in RIPA buffer supplemented with protease and phosphatase inhibitor cocktail (Beyotime) for 30 min. The soluble fractions were separated from pellets after centrifugation at 15,000 rpm for 15 min. The pellets were washed three times with PBS containing 1% Triton X-100 and protease inhibitor, and further solubilized in 6 M Guanidine hydrochloride (GdnHCl) containing 50 mM Tris-HCl (pH 8.0), 200 mM NaCl and protease inhibitor for 1 h at 4 °C. After centrifugation at 15,000 rpm for 15 min, the Triton X-100-insoluble fractions were collected from the supernatants. The soluble and insoluble fractions were boiled in sodium dodecyl sulfate (SDS) sample buffer for 10 min and used for SDS-polyacrylamide gel electrophoresis (SDS-PAGE) and immunoblotting analysis.

Collected primordial follicle oocytes were mixed with SDS sample buffer and boiled for 5 min at 100 °C for SDS-PAGE and immunoblotting analysis. Western blot was performed as described previously [65]. In brief, equal amounts of total protein were separated in a 4–15% SDS-PAGE gel and transferred onto PVDF membranes. After blocking with 5% BSA for 2 h at room temperature, the membranes were incubated with diluted primary antibodies at 4 °C overnight. Then, the membranes were washed three times with TBST and incubated with horseradish peroxidase-conjugated secondary antibodies. The signals were detected with SuperSignal West Dura Extended Duration Substrate (Thermo Fisher Scientific), imaged with a LAS-3000, and analyzed with Quantity One software (Bio-Rad Laboratories).

The dilutions of primary antibodies were as follows: antibodies against PPP4C, p-AKT^S473^, p-mTOR^S2448^, p-MAPKAPK-2^T334^, anti-p-S6K ^T389^, p-rpS6^S240/244^ and p62 at 1:1000 and antibodies against Ub-H2A, β-actin, GAPDH and α-tubulin at 1:2000.

### Transmission electron microscopy

The ovaries were fixed with 2.5% glutaraldehyde and 2% PFA overnight at 4 °C. After fixation with 1% OsO4 in 0.2 M cacodylate buffer, the tissues were dehydrated and embedded in resin. Ultrathin sections were cut on an ultramicrotome, stained with uranyl acetate and lead citrate and observed under a Spirit 120 kV transmission electron microscope (FEI, USA).

### Statistical analyses

All experiments were repeated at least three times. Student’s *t*-test was used for statistical analysis and performed using SPSS. Data were expressed as mean ± SEM and values are statistically significant at *P < 0.05; **P < 0.01; ***P < 0.001.

## Supplementary information

Supplemental Material

## Data Availability

The main data generated or analyzed during this study are included in this published article and its Supplementary Information files. The datasets generated or analyzed during this study can be obtained from the corresponding author upon reasonable request.
