# Supplementary material for: Protein phosphatase 4 maintains the survival of primordial follicles by regulating autophagy in oocytes
Source: Cell Death Dis. 2024 Sep 8;15(9):658. doi: 10.1038/s41419-024-07051-4 (PMC11381532; doi:10.1038/s41419-024-07051-4)
Supplement: Supplementary file 1 — Supplemental Material [file 41419_2024_7051_MOESM1_ESM.pdf]

Supplementary

Protein phosphatase 4 maintains the survival of primordial follicles by regulating autophagy in oocytes

Ming-Zhe Dong[1, 2], Ying-Chun Ouyang[1, 2], Shi-Cai Gao[1, 2], Lin-Jian Gu[1, 2], Jia-Ni Guo[1, 2], Si-Min Sun[1, 2], Zhen-Bo Wang[1, 2] and Qing-Yuan Sun[3, *]

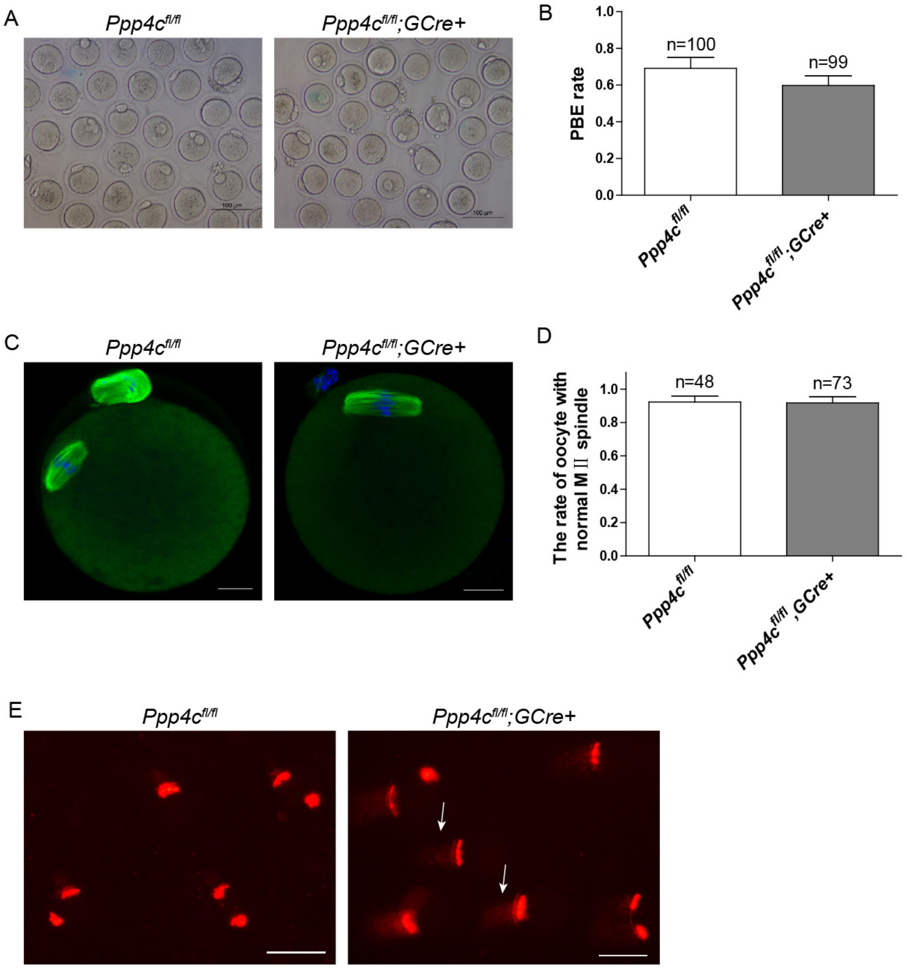

Fig S1. Depletion of PPP4C impairs genomic integrity in oocytes but does not affect oocyte meiotic maturation. (**A-B**) Comparable PBE rates in *Ppp4c$^{fl/fl}$* and *Ppp4c$^{fl/fl}$;GCre+* oocytes. Germinal vesicle (GV) oocytes were isolated and matured in

vitro; oocytes that extruded the first polar body (PBE) were counted at 14 h. Representative DIC images are shown. Data are presented as mean ± s.e.m. Scale bars: 100 μm. (**C-D**) Representative images of staining for DNA (blue) and immunostaining for α-tubulin (green) showing normal spindle assembly in *Ppp4c*$^{fl/fl}$ and *Ppp4c*$^{fl/fl}$*;GCre+* oocytes at the M II stage. Scale bars: 20 μm. The percentages of oocytes with a normal spindle at the M II stage of each genotype are presented as mean ± s.e.m. The numbers of analyzed oocytes are indicated (n). (**E**) M II oocytes of *Ppp4c*$^{fl/fl}$ and *Ppp4c*$^{fl/fl}$*;GCre+* females were analyzed by single-cell gel electrophoresis (comet assay). Scale bars: 100 μm. Each experiment was repeated at least 3 times.

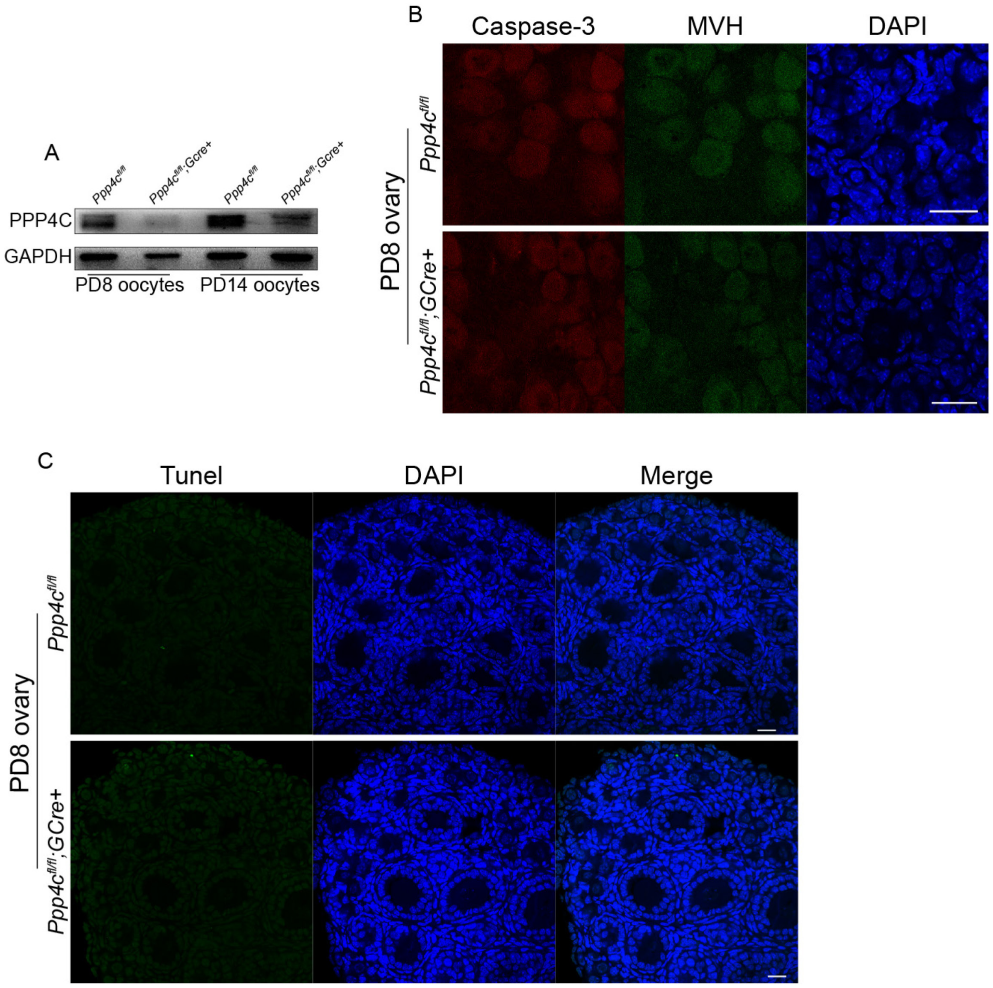

Fig S2. Depletion of PPP4C does not induce apoptosis in primordial follicle

oocytes. (**A**) Western blots showing the deletion of *Ppp4c* in PD8 and PD14 oocytes. Level of GAPDH was detected as internal control. (**B**) Immunofluorescent staining of PD8 ovarian sections showing no significant difference of Caspase-3 between *Ppp4c*$^{fl/fl}$ and *Ppp4c*$^{fl/fl}$*;GCre+* ovaries. Red: Caspase-3; Green: MVH; Blue: DAPI. Scale bars: 20 μm. (**C**) Immunofluorescent staining of PD8 ovarian sections showing no significant difference of Tunel staining between *Ppp4c*$^{fl/fl}$ and *Ppp4c*$^{fl/fl}$*;GCre+* ovaries. Green: Tunel; Blue: DAPI. Scale bars: 20 μm. Each experiment was repeated at least 3 times.

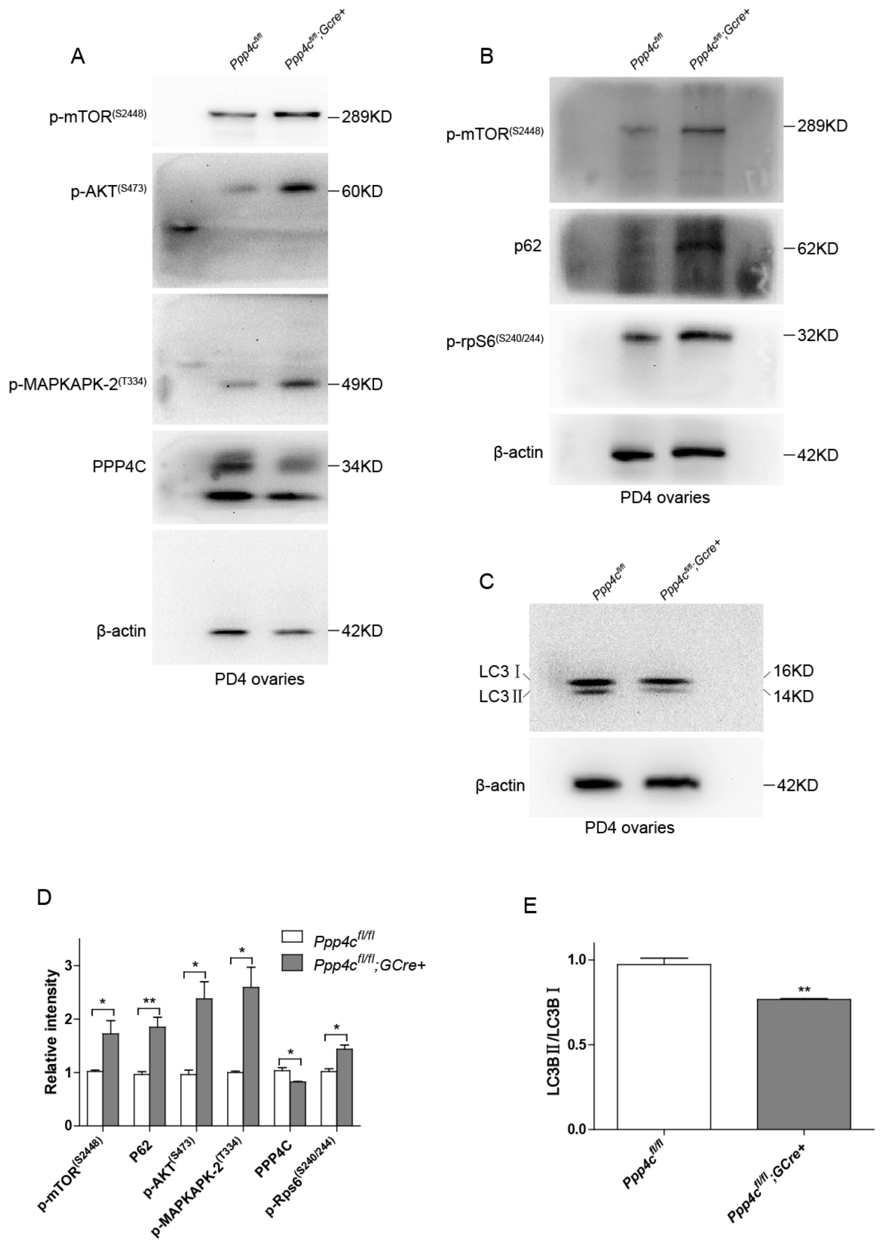

Fig S3. Full and uncropped western blots of *Ppp4c*$^{fl/fl}$ and *Ppp4c*$^{fl/fl}$*;GCre*+ ovaries at PD4. (**A-C**) Full and uncropped western blots showing levels of p-mTOR^{S2448}, p-AKT^{S473}, p-MAPKAPK-2^{T334}, PPP4C, p-rpS6$^{S240/244}$, P62 and LC3B. Level of β-actin was detected as internal control. (**D**) Quantification of proteins based on A-B. (**E**) Quantification of LC3II/I based on C. Data are presented as mean ± s.e.m. *P < 0.05, * *P < 0.01, * * *P < 0.001. Each experiment was repeated at least 3 times.

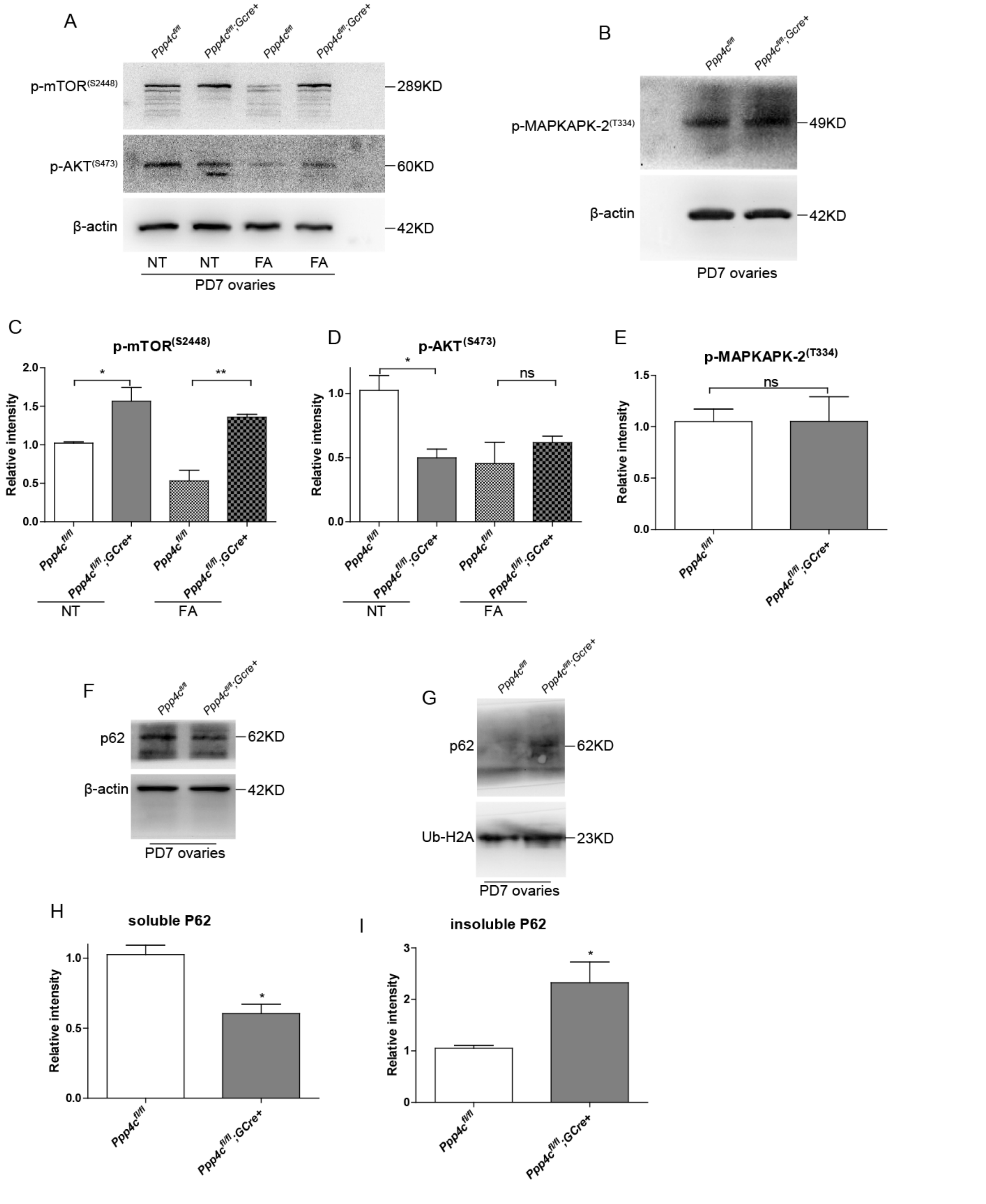

Fig S4. Full and uncropped western blots of *Ppp4c$^{fl/fl}$* and *Ppp4c$^{fl/fl}$;GCre+* ovaries at PD7. (**A-B**) Full and uncropped western blots showing levels of p-mTOR^{S2448}, p-AKT^{S473} and p-MAPKAPK-2^{T334}. Mice were treated with (FA) or without (NT) fasting for 2 days before sacrifice. Level of β-actin was detected as internal control. (**C-E**) Quantification of proteins based on A-B. (**F**) Full and uncropped western blot analysis of p62 in soluble lysates of PD7 ovaries. Level of β-actin was detected as internal control. (**G**) Full and

uncropped western blot analysis of p62 in insoluble lysates of PD7 ovaries. Level of Ub-H2A was detected as internal control. (**H-I**) Quantification of p62 based on F-G. Data are presented as mean ± s.e.m. *P < 0.05, * *P < 0.01. Each experiment was repeated at least 3 times.

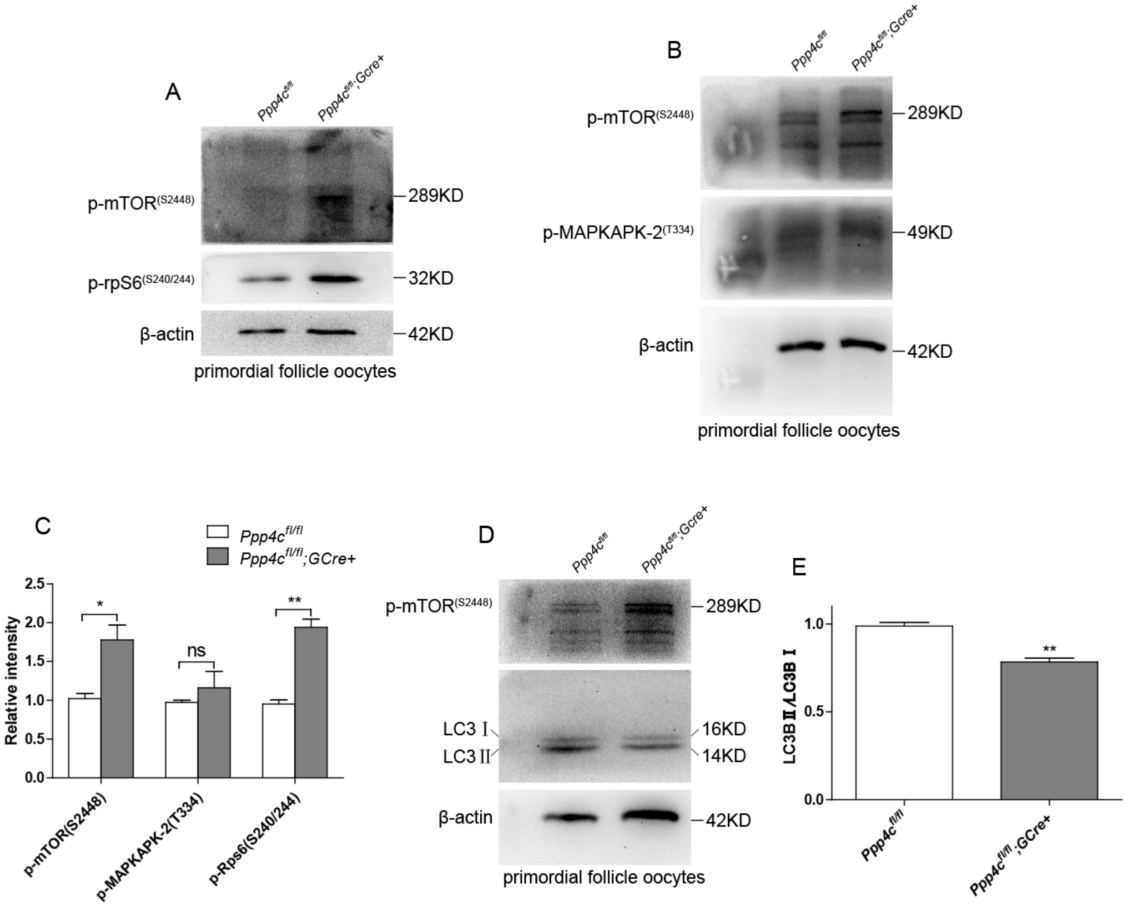

Fig S5. Full and uncropped western blots of *Ppp4c$^{fl/fl}$* and *Ppp4c$^{fl/fl}$;GCre*+ primordial follicle oocytes at PD7. (**A, B and D**) Full and uncropped western blots showing levels of p-mTOR^{S2448}, p-MAPKAPK-2^{T334}, p-rpS6$^{S240/244}$ and LC3B. Level of β-actin was detected as internal control. (**C**) Quantification of proteins based on A-B. (**E**) Quantification of LC3II/I based on D. Data are presented as mean ± s.e.m. *P < 0.05, * * *P < 0.001. Each experiment was repeated at least 3 times.

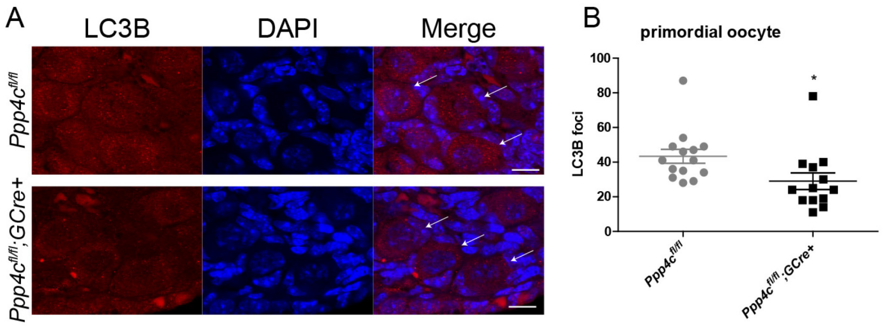

Fig S6. Autophagic flux is disrupted in the primordial follicle oocytes. (**A**) Representative Immunofluorescent staining images of PD5 ovarian sections showing decreased LC3 foci in primordial follicle oocytes of *Ppp4c*$^{fl/fl}$*;GCre+*. White arrow points to the primordial follicle. Scale bars: 10 μm. (**B**) Quantification of LC3B foci in A was analyzed with ImageJ software. Data are presented as mean ± s.e.m. *$P < 0.05$. Each experiment was repeated at least 3 times.

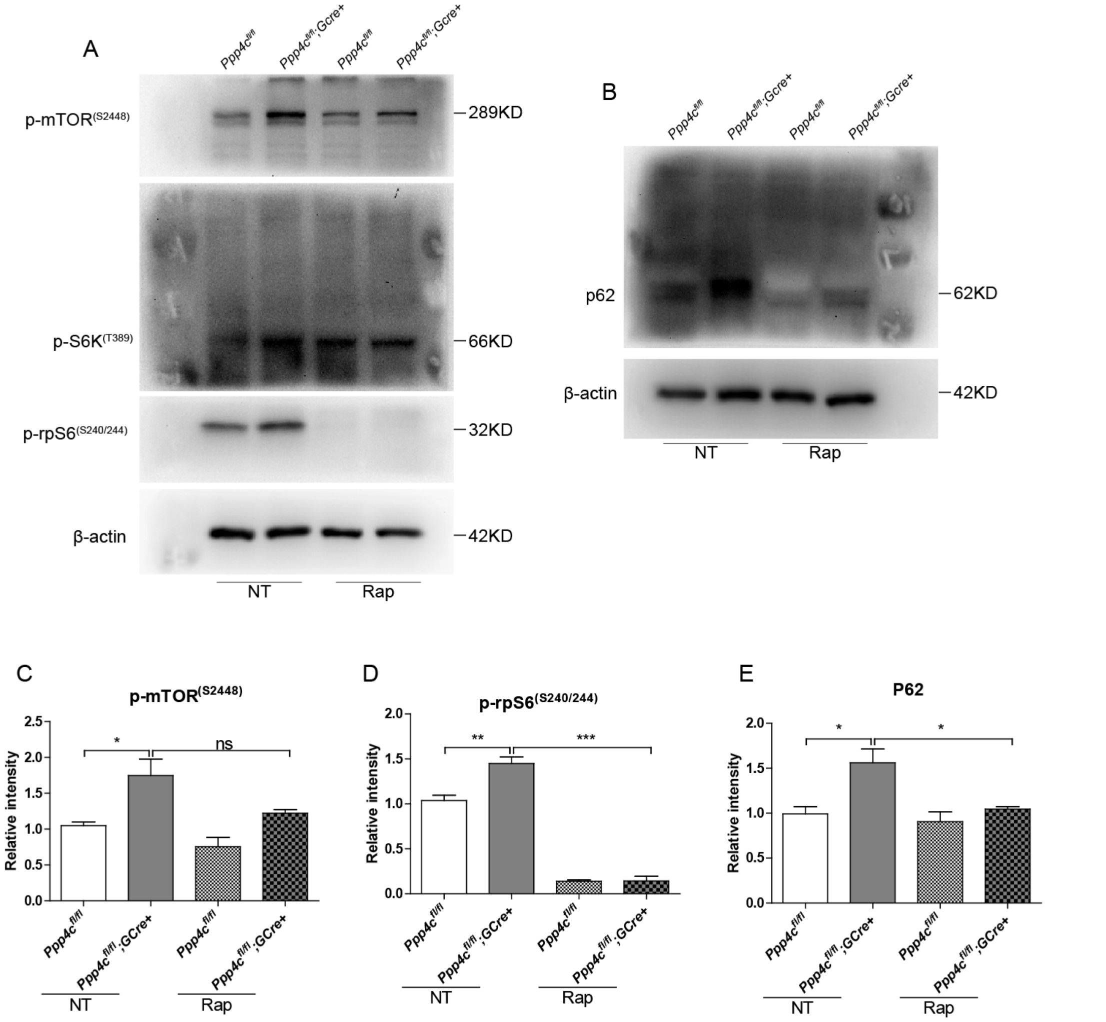

Fig S7. Full and uncropped western blots of *Ppp4c$^{fl/fl}$* and *Ppp4c$^{fl/fl}$;GCre+* ovaries at PD4. (**A-B**) Full and uncropped western blots showing levels of p-mTOR^{S2448}, p-S6K^{S389}, p-rpS6$^{S240/244}$ and P62. Level of β-actin was detected as internal control. (**C-E**) Quantification of proteins based on A-B. Data are presented as mean ± s.e.m. *P < 0.05, * * *P < 0.001. Each experiment was repeated at least 3 times.

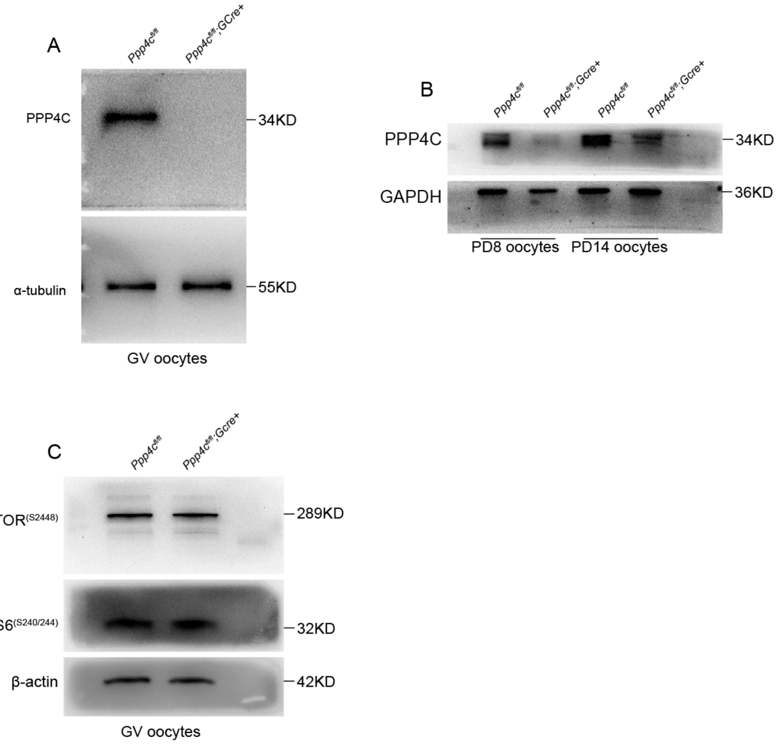

Fig S8. Full and uncropped western blots. (**A**) Full and uncropped western blots showing levels of PPP4C in GV oocytes. Level of α-tubulin was detected as internal control. (**B**) Full and uncropped western blots showing levels of PPP4C in PD8 and PD14 oocytes. Level of GAPDH was detected as internal control. (**C**) Full and uncropped western blots showing levels of p-mTOR^{S2448} and p-rpS6$^{S240/244}$ in GV oocytes. Level of β-actin was detected as internal control.
